# Preaustinoid A: a meroterpene produced by *Penicillium sp*.

**DOI:** 10.1107/S1600536808043481

**Published:** 2009-01-08

**Authors:** Stella H. Maganhi, Taicia Pacheco Fill, Edson Rodrigues-Fo, Ignez Caracelli, Julio Zukerman-Schpector

**Affiliations:** a Department of Chemistry, Universidade Federal de São Carlos, 13565-905 São Carlos, SP, Brazil; b BioMat - Physics Department, Universidade Estadual Paulista "Júlio de Mesquita Filho", UNESP, 17033-360 Bauru, SP, Brazil

## Abstract

The title meroterpene preaustinoid A (systematic name: methyl 15-hydr­oxy-2,6,6,10,13,15-hexa­methyl-17-methyl­ene-7,14,16-trioxotetra­cyclo­[11.3.1.0^2,11^.0^5,10^]hepta­decane-1-car­box­yl­ate), C_26_H_36_O_6_, features a fused four-ring arrangement. Three rings are in different distorted chair conformations and the other is in a distorted boat conformation. The absolute configuration was established based on [α_D_] = −4.97° (*c* = 1.10 g l^−1^, CH_2_Cl_2_). In the crystal, the mol­ecules are connected into supra­molecular chains *via* O—H⋯O hydrogen bonds.

## Related literature

For related literature, see: dos Santos & Rodrigues-Fo (2002 ▶). For structure analysis, see: Cremer and Pople (1975 ▶); Iulek and Zukerman-Schpector (1997 ▶)).
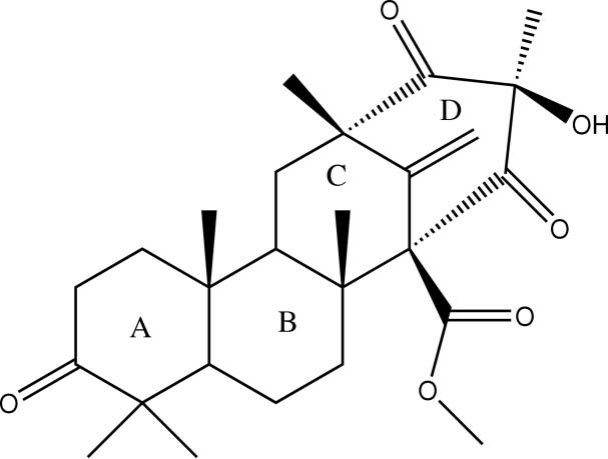


## Experimental

### 

#### Crystal data

C_26_H_36_O_6_
*M* *_r_* = 444.55
Orthorhombic, 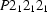

*a* = 8.5023 (2) Å
*b* = 13.5405 (2) Å
*c* = 19.7127 (4) Å
*V* = 2269.43 (8) Å^3^
*Z* = 4
Mo *K*α radiation
μ = 0.09 mm^−1^
*T* = 290 (2) K
0.37 × 0.28 × 0.11 mm

#### Data collection

Bruker APEXII CCD area-detector diffractometer
Absorption correction: none
27134 measured reflections
2938 independent reflections
2677 reflections with *I* > 2σ(*I*)
*R*_int_ = 0.034

#### Refinement

*R*[*F* ^2^ > 2σ(*F* ^2^)] = 0.036
*wR*(*F* ^2^) = 0.093
*S* = 1.03
2938 reflections
297 parameters
H-atom parameters constrained
Δρ_max_ = 0.22 e Å^−3^
Δρ_min_ = −0.13 e Å^−3^

### 

Data collection: *APEX2*, *COSMO* and *BIS* (Bruker, 2006 ▶); cell refinement: *SAINT* (Bruker, 2006 ▶); data reduction: *SAINT*; program(s) used to solve structure: *SIR97* (Altomare *et al.*, 1999 ▶); program(s) used to refine structure: *SHELXL97* (Sheldrick, 2008 ▶); molecular graphics: *ORTEP-3* (Farrugia, 1997 ▶); software used to prepare material for publication: *WinGX* (Farrugia, 1999 ▶).

## Supplementary Material

Crystal structure: contains datablocks global, I. DOI: 10.1107/S1600536808043481/tk2346sup1.cif

Structure factors: contains datablocks I. DOI: 10.1107/S1600536808043481/tk2346Isup2.hkl

Additional supplementary materials:  crystallographic information; 3D view; checkCIF report

## Figures and Tables

**Table 1:** Hydrogen-bond geometry (Å, °)

*D*—H⋯*A*	*D*—H	H⋯*A*	*D*⋯*A*	*D*—H⋯*A*
O5—H1*O*5⋯O3^i^	0.82	2.05	2.870 (2)	173

Symmetry code: (i) 
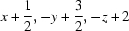
.

**Fig. 1.**
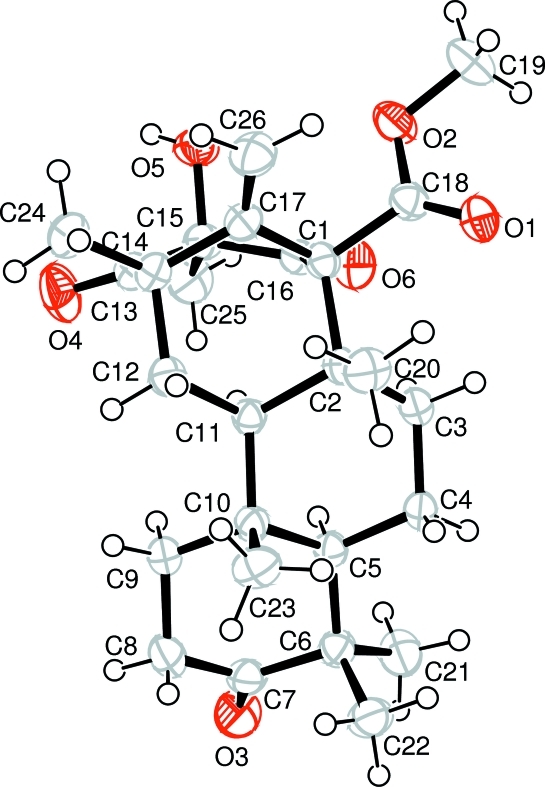
The molecular structure of (I) showing atom labelling scheme and displacement ellipsoids at the 30% probability level (arbitrary spheres for the H atoms).

## supplementary crystallographic information

### Comment

Endophytic fungi have been a rich source of important biologically active secondary metabolites, in particular meroterpenoids, a class of complex metabolites derived from a mixed terpenoid-polyketide biosynthetic pathway. During an on-going study of substances produced by endophytic fungi, the title compound (I) was isolated and its structure postulated based on APCIMS (Atmospheric Pressure Chemical Ionization Mass Spectrometry), HREIMS (High Resolution Electrospray Mass Spectrometry) and a variety of NMR studies (dos Santos and Rodrigues-Fo, 2002). As suitable crystals were subsequently obtained, a crystal structure determination was undertaken. The four fused rings are in different distorted conformations. Rings A and C are distorted towards a half-chair conformation, ring B is distorted towards a half-boat conformation, and ring D is a boat conformation that is highly distorted towards a half-boat. The ring-puckering parameters (Cremer & Pople, 1975, Iulek & Zukerman-Shpector, 1997) for rings A,B,*C*, D are: q_2_ = 0.062 (2), 0.093 (2), 0.059 (2), 0.582 (2) Å, q_3_ = -0.524 (2), 0.577 (2), -0.624 (2), -0.139 (2) Å, Q = 0.528 (2), 0.584 (2), 0.627 (2), 0.599 (2)°, φ_2_ = 90 (2), 172 (1), -149 (2), -122.4 (2)°, and θ_2_ = 173.3 (2), 9.1 (2), 174.6 (2), 103.4 (2)°, respectively. The absolute configuration was established based on the [α_D_] = -4.97° (c 1.10 g/L, CH_2_Cl_2_) and the results reported in dos Santos & Rodrigues-Fo (2002). The molecules are linked into a supramolecular chain *via* intermolecular O-H···O hydrogen bonds, Table 1.

### Experimental

Compound (I), Preaustinoid A, was produced during cultivation of *Penicillum sp* over sterilized rice, and isolated from the methanol extract of the culture. Suitable crystals were obtained, by slow evaporation, from a mixture of dichloromethane, methanol and water.

### Refinement

In the absence of significant anomalous scattering effects, Friedel pairs were averaged in the final refinement. The H atoms were refined in the riding-model approximation with C—H = 0.93 - 0.98 Å and (0.82 for O—H), and with *U*_iso_(H) = 1.5*U*_eq_(methyl-C) or 1.2*U*_eq_(remaining-C and –O).

### Crystal data

**Table:**

C_26_H_36_O_6_	*F*(000) = 960
*M**_r_* = 444.55	*D*_x_ = 1.301 Mg m^−^^3^
Orthorhombic, *P*2_1_2_1_2_1_	Mo *K*α radiation, λ = 0.71073 Å
Hall symbol: P 2ac 2ab	Cell parameters from 22936 reflections
*a* = 8.5023 (2) Å	θ = 1.0–27.5°
*b* = 13.5405 (2) Å	µ = 0.09 mm^−^^1^
*c* = 19.7127 (4) Å	*T* = 290 K
*V* = 2269.43 (8) Å^3^	Prism, colorless
*Z* = 4	0.37 × 0.28 × 0.11 mm

### Data collection

**Table:**

Bruker APEXII CCD area-detector diffractometer	2677 reflections with *I* > 2σ(*I*)
Radiation source: fine-focus sealed tube	*R*_int_ = 0.034
graphite	θ_max_ = 27.5°, θ_min_ = 1.8°
φ and ω scans	*h* = −11→9
27134 measured reflections	*k* = −17→17
2938 independent reflections	*l* = −25→25

### Refinement

**Table:**

Refinement on *F*^2^	Secondary atom site location: difference Fourier map
Least-squares matrix: full	Hydrogen site location: inferred from neighbouring sites
*R*[*F*^2^ > 2σ(*F*^2^)] = 0.036	H-atom parameters constrained
*wR*(*F*^2^) = 0.093	*w* = 1/[σ^2^(*F*_o_^2^) + (0.0531*P*)^2^ + 0.3814*P*] where *P* = (*F*_o_^2^ + 2*F*_c_^2^)/3
*S* = 1.03	(Δ/σ)_max_ < 0.001
2938 reflections	Δρ_max_ = 0.22 e Å^−^^3^
297 parameters	Δρ_min_ = −0.13 e Å^−^^3^
0 restraints	Absolute structure: [α_D_] = -4.97° and results in dos Santos & Rodrigues-Fo (2002)
Primary atom site location: structure-invariant direct methods	

### Special details

**Table:**

Geometry. All e.s.d.'s (except the e.s.d. in the dihedral angle between two l.s. planes) are estimated using the full covariance matrix. The cell e.s.d.'s are taken into account individually in the estimation of e.s.d.'s in distances, angles and torsion angles; correlations between e.s.d.'s in cell parameters are only used when they are defined by crystal symmetry. An approximate (isotropic) treatment of cell e.s.d.'s is used for estimating e.s.d.'s involving l.s. planes.
Refinement. Refinement of *F*^2^ against ALL reflections. The weighted *R*-factor *wR* and goodness of fit *S* are based on *F*^2^, conventional *R*-factors *R* are based on *F*, with *F* set to zero for negative *F*^2^. The threshold expression of *F*^2^ > σ(*F*^2^) is used only for calculating *R*-factors(gt) *etc*. and is not relevant to the choice of reflections for refinement. *R*-factors based on *F*^2^ are statistically about twice as large as those based on *F*, and *R*- factors based on ALL data will be even larger.

### Fractional atomic coordinates and isotropic or equivalent isotropic displacement parameters (Å^2^)

**Table:**

	*x*	*y*	*z*	*U*_iso_*/*U*_eq_	
C1	0.6598 (2)	1.07289 (12)	1.05643 (9)	0.0288 (4)	
C2	0.6226 (2)	1.08110 (12)	0.97653 (9)	0.0285 (4)	
C3	0.4489 (2)	1.10698 (14)	0.96387 (10)	0.0339 (4)	
H3A	0.3835	1.0666	0.9931	0.041*	
H3B	0.4314	1.1756	0.9760	0.041*	
C4	0.3986 (2)	1.09105 (13)	0.89014 (10)	0.0335 (4)	
H4A	0.4588	1.1343	0.8607	0.040*	
H4B	0.2882	1.1076	0.8851	0.040*	
C5	0.4252 (2)	0.98347 (13)	0.86909 (9)	0.0281 (4)	
H5	0.3789	0.9444	0.9059	0.034*	
C6	0.3310 (2)	0.95241 (13)	0.80444 (10)	0.0333 (4)	
C7	0.3688 (3)	0.84397 (14)	0.78946 (10)	0.0375 (4)	
C8	0.5381 (3)	0.81430 (17)	0.79148 (13)	0.0480 (5)	
H8A	0.5925	0.8437	0.7532	0.058*	
H8B	0.5456	0.7431	0.7871	0.058*	
C9	0.6192 (3)	0.84641 (14)	0.85749 (10)	0.0379 (4)	
H9A	0.7299	0.8294	0.8551	0.045*	
H9B	0.5736	0.8103	0.8952	0.045*	
C10	0.6034 (2)	0.95805 (13)	0.87113 (9)	0.0289 (4)	
C11	0.6583 (2)	0.97663 (12)	0.94619 (8)	0.0266 (3)	
H11	0.5967	0.9304	0.9737	0.032*	
C12	0.8309 (2)	0.94807 (15)	0.95867 (10)	0.0346 (4)	
H12A	0.8986	0.9948	0.9355	0.041*	
H12B	0.8503	0.8833	0.9394	0.041*	
C13	0.8741 (2)	0.94642 (14)	1.03528 (10)	0.0338 (4)	
C14	0.7714 (3)	0.86428 (14)	1.06479 (10)	0.0372 (4)	
C15	0.6277 (3)	0.89190 (13)	1.10736 (9)	0.0346 (4)	
C16	0.5554 (2)	0.99228 (14)	1.08868 (9)	0.0322 (4)	
C17	0.8335 (2)	1.04648 (13)	1.06521 (9)	0.0317 (4)	
C18	0.6196 (2)	1.16872 (13)	1.09524 (10)	0.0341 (4)	
C19	0.5811 (3)	1.23338 (17)	1.20486 (12)	0.0498 (6)	
H19A	0.4780	1.2564	1.1928	0.075*	
H19B	0.6558	1.2858	1.1989	0.075*	
H19C	0.5811	1.2126	1.2514	0.075*	
C20	0.7279 (3)	1.16296 (14)	0.94607 (10)	0.0392 (5)	
H20A	0.7083	1.2241	0.9692	0.059*	
H20B	0.7043	1.1706	0.8987	0.059*	
H20C	0.8364	1.1450	0.9513	0.059*	
C21	0.1540 (3)	0.96145 (17)	0.81845 (13)	0.0475 (5)	
H21A	0.1281	0.9253	0.8588	0.071*	
H21B	0.0961	0.9349	0.7808	0.071*	
H21C	0.1270	1.0297	0.8245	0.071*	
C22	0.3678 (3)	1.01321 (16)	0.74011 (10)	0.0448 (5)	
H22A	0.3552	1.0822	0.7498	0.067*	
H22B	0.2969	0.9944	0.7044	0.067*	
H22C	0.4741	1.0008	0.7261	0.067*	
C23	0.7051 (2)	1.01368 (17)	0.81888 (10)	0.0409 (5)	
H23A	0.7011	0.9798	0.7761	0.061*	
H23B	0.8119	1.0163	0.8346	0.061*	
H23C	0.6656	1.0796	0.8134	0.061*	
C24	1.0483 (3)	0.91813 (19)	1.04234 (13)	0.0496 (6)	
H24A	1.0757	0.9149	1.0895	0.074*	
H24B	1.1122	0.9669	1.0203	0.074*	
H24C	1.0658	0.8549	1.0216	0.074*	
C25	0.5021 (3)	0.81210 (15)	1.10693 (14)	0.0515 (6)	
H25A	0.4182	0.8306	1.1369	0.077*	
H25B	0.5471	0.7508	1.1218	0.077*	
H25C	0.4616	0.8045	1.0618	0.077*	
C26	0.9386 (3)	1.10751 (16)	1.09100 (11)	0.0429 (5)	
H26A	1.0443	1.0901	1.0915	0.051*	
H26B	0.9069	1.1680	1.1087	0.051*	
O1	0.5918 (2)	1.24745 (10)	1.07174 (8)	0.0472 (4)	
O2	0.6234 (2)	1.15134 (10)	1.16213 (7)	0.0466 (4)	
O3	0.2674 (2)	0.78454 (11)	0.77637 (9)	0.0544 (4)	
O4	0.8037 (2)	0.77818 (11)	1.05593 (10)	0.0617 (5)	
O5	0.6898 (2)	0.90860 (11)	1.17396 (7)	0.0485 (4)	
H1O5	0.7163	0.8558	1.1907	0.058*	
O6	0.41897 (17)	1.00801 (12)	1.10126 (8)	0.0465 (4)	

### Atomic displacement parameters (Å^2^)

**Table:**

	*U*^11^	*U*^22^	*U*^33^	*U*^12^	*U*^13^	*U*^23^
C1	0.0316 (9)	0.0236 (7)	0.0311 (8)	−0.0013 (7)	−0.0004 (7)	−0.0008 (6)
C2	0.0322 (9)	0.0238 (7)	0.0295 (8)	0.0008 (7)	−0.0008 (7)	0.0006 (6)
C3	0.0352 (10)	0.0299 (8)	0.0365 (9)	0.0057 (8)	−0.0024 (8)	−0.0049 (7)
C4	0.0348 (9)	0.0282 (8)	0.0375 (9)	0.0073 (8)	−0.0057 (8)	−0.0025 (7)
C5	0.0288 (9)	0.0265 (8)	0.0291 (8)	0.0009 (7)	0.0010 (7)	0.0010 (6)
C6	0.0334 (9)	0.0303 (8)	0.0362 (9)	0.0002 (8)	−0.0034 (8)	−0.0013 (7)
C7	0.0468 (12)	0.0338 (9)	0.0319 (9)	−0.0007 (9)	−0.0064 (9)	−0.0024 (7)
C8	0.0513 (13)	0.0410 (11)	0.0516 (13)	0.0118 (10)	−0.0063 (11)	−0.0160 (10)
C9	0.0407 (11)	0.0352 (9)	0.0378 (10)	0.0111 (9)	−0.0046 (9)	−0.0064 (8)
C10	0.0282 (9)	0.0300 (8)	0.0285 (8)	0.0025 (7)	0.0025 (7)	0.0004 (7)
C11	0.0268 (8)	0.0265 (8)	0.0265 (8)	0.0016 (7)	0.0018 (7)	0.0012 (6)
C12	0.0299 (9)	0.0406 (10)	0.0331 (9)	0.0070 (8)	0.0009 (8)	−0.0012 (8)
C13	0.0309 (9)	0.0345 (9)	0.0360 (9)	0.0056 (8)	−0.0044 (8)	−0.0009 (8)
C14	0.0451 (11)	0.0336 (9)	0.0329 (9)	0.0053 (9)	−0.0056 (9)	0.0001 (7)
C15	0.0445 (11)	0.0279 (8)	0.0313 (9)	−0.0043 (8)	−0.0031 (8)	0.0014 (7)
C16	0.0373 (10)	0.0308 (9)	0.0284 (8)	−0.0032 (8)	0.0007 (8)	−0.0023 (7)
C17	0.0331 (9)	0.0318 (8)	0.0302 (8)	0.0006 (8)	−0.0011 (8)	0.0027 (7)
C18	0.0344 (10)	0.0303 (8)	0.0376 (10)	−0.0015 (8)	−0.0026 (8)	−0.0041 (7)
C19	0.0606 (15)	0.0438 (11)	0.0450 (11)	−0.0037 (11)	0.0107 (11)	−0.0153 (10)
C20	0.0450 (12)	0.0331 (9)	0.0396 (10)	−0.0096 (9)	−0.0007 (9)	0.0069 (8)
C21	0.0339 (11)	0.0461 (11)	0.0624 (14)	−0.0015 (9)	−0.0053 (10)	−0.0104 (10)
C22	0.0533 (13)	0.0453 (11)	0.0358 (10)	0.0002 (10)	−0.0068 (10)	0.0038 (9)
C23	0.0356 (10)	0.0544 (12)	0.0328 (9)	−0.0030 (9)	0.0050 (8)	0.0065 (9)
C24	0.0362 (11)	0.0583 (13)	0.0543 (13)	0.0131 (11)	−0.0111 (10)	−0.0098 (11)
C25	0.0584 (14)	0.0319 (10)	0.0642 (14)	−0.0127 (10)	−0.0048 (12)	0.0048 (10)
C26	0.0375 (11)	0.0417 (11)	0.0496 (12)	−0.0050 (9)	−0.0073 (9)	−0.0005 (9)
O1	0.0638 (10)	0.0288 (6)	0.0490 (8)	0.0075 (7)	−0.0084 (8)	−0.0050 (6)
O2	0.0698 (11)	0.0342 (7)	0.0359 (7)	0.0028 (7)	0.0037 (8)	−0.0064 (6)
O3	0.0614 (11)	0.0355 (8)	0.0664 (10)	−0.0091 (8)	−0.0145 (9)	−0.0019 (7)
O4	0.0825 (13)	0.0312 (7)	0.0716 (11)	0.0123 (8)	0.0137 (10)	−0.0002 (7)
O5	0.0746 (12)	0.0382 (7)	0.0327 (7)	0.0031 (8)	−0.0117 (7)	−0.0005 (6)
O6	0.0388 (8)	0.0467 (8)	0.0539 (9)	−0.0008 (7)	0.0106 (7)	0.0049 (7)

### Geometric parameters (Å, °)

**Table:**

C1—C17	1.529 (3)	C13—C24	1.536 (3)
C1—C16	1.544 (3)	C14—O4	1.210 (2)
C1—C18	1.545 (2)	C14—C15	1.529 (3)
C1—C2	1.610 (2)	C15—O5	1.433 (2)
C2—C3	1.538 (3)	C15—C25	1.519 (3)
C2—C20	1.546 (3)	C15—C16	1.536 (3)
C2—C11	1.566 (2)	C16—O6	1.205 (2)
C3—C4	1.530 (3)	C17—C26	1.319 (3)
C3—H3A	0.9700	C18—O1	1.186 (2)
C3—H3B	0.9700	C18—O2	1.340 (2)
C4—C5	1.531 (2)	C19—O2	1.440 (2)
C4—H4A	0.9700	C19—H19A	0.9600
C4—H4B	0.9700	C19—H19B	0.9600
C5—C10	1.554 (2)	C19—H19C	0.9600
C5—C6	1.563 (3)	C20—H20A	0.9600
C5—H5	0.9800	C20—H20B	0.9600
C6—C7	1.532 (3)	C20—H20C	0.9600
C6—C21	1.535 (3)	C21—H21A	0.9600
C6—C22	1.544 (3)	C21—H21B	0.9600
C7—O3	1.207 (3)	C21—H21C	0.9600
C7—C8	1.495 (3)	C22—H22A	0.9600
C8—C9	1.536 (3)	C22—H22B	0.9600
C8—H8A	0.9700	C22—H22C	0.9600
C8—H8B	0.9700	C23—H23A	0.9600
C9—C10	1.541 (2)	C23—H23B	0.9600
C9—H9A	0.9700	C23—H23C	0.9600
C9—H9B	0.9700	C24—H24A	0.9600
C10—C23	1.541 (3)	C24—H24B	0.9600
C10—C11	1.572 (2)	C24—H24C	0.9600
C11—C12	1.538 (3)	C25—H25A	0.9600
C11—H11	0.9800	C25—H25B	0.9600
C12—C13	1.554 (3)	C25—H25C	0.9600
C12—H12A	0.9700	C26—H26A	0.9300
C12—H12B	0.9700	C26—H26B	0.9300
C13—C17	1.518 (3)	O5—H1O5	0.8200
C13—C14	1.529 (3)		
			
C17—C1—C16	110.07 (14)	C17—C13—C24	114.00 (17)
C17—C1—C18	110.75 (15)	C14—C13—C24	109.54 (17)
C16—C1—C18	105.22 (15)	C17—C13—C12	108.14 (15)
C17—C1—C2	108.47 (15)	C14—C13—C12	104.21 (16)
C16—C1—C2	109.79 (14)	C24—C13—C12	108.61 (17)
C18—C1—C2	112.51 (14)	O4—C14—C13	121.1 (2)
C3—C2—C20	109.24 (15)	O4—C14—C15	119.7 (2)
C3—C2—C11	109.27 (15)	C13—C14—C15	119.16 (16)
C20—C2—C11	112.77 (15)	O5—C15—C25	112.11 (17)
C3—C2—C1	111.30 (15)	O5—C15—C14	104.29 (17)
C20—C2—C1	108.39 (14)	C25—C15—C14	112.63 (17)
C11—C2—C1	105.86 (13)	O5—C15—C16	103.14 (14)
C4—C3—C2	112.98 (16)	C25—C15—C16	110.30 (17)
C4—C3—H3A	109.0	C14—C15—C16	113.86 (16)
C2—C3—H3A	109.0	O6—C16—C15	119.46 (18)
C4—C3—H3B	109.0	O6—C16—C1	120.88 (18)
C2—C3—H3B	109.0	C15—C16—C1	119.63 (17)
H3A—C3—H3B	107.8	C26—C17—C13	123.72 (19)
C3—C4—C5	110.50 (15)	C26—C17—C1	123.45 (18)
C3—C4—H4A	109.6	C13—C17—C1	112.61 (16)
C5—C4—H4A	109.6	O1—C18—O2	123.17 (17)
C3—C4—H4B	109.6	O1—C18—C1	127.27 (18)
C5—C4—H4B	109.6	O2—C18—C1	109.54 (15)
H4A—C4—H4B	108.1	O2—C19—H19A	109.5
C4—C5—C10	110.34 (15)	O2—C19—H19B	109.5
C4—C5—C6	113.65 (15)	H19A—C19—H19B	109.5
C10—C5—C6	117.46 (15)	O2—C19—H19C	109.5
C4—C5—H5	104.6	H19A—C19—H19C	109.5
C10—C5—H5	104.6	H19B—C19—H19C	109.5
C6—C5—H5	104.6	C2—C20—H20A	109.5
C7—C6—C21	108.47 (17)	C2—C20—H20B	109.5
C7—C6—C22	108.08 (17)	H20A—C20—H20B	109.5
C21—C6—C22	107.68 (18)	C2—C20—H20C	109.5
C7—C6—C5	107.91 (15)	H20A—C20—H20C	109.5
C21—C6—C5	109.53 (17)	H20B—C20—H20C	109.5
C22—C6—C5	114.99 (16)	C6—C21—H21A	109.5
O3—C7—C8	120.95 (19)	C6—C21—H21B	109.5
O3—C7—C6	122.0 (2)	H21A—C21—H21B	109.5
C8—C7—C6	117.03 (18)	C6—C21—H21C	109.5
C7—C8—C9	112.26 (18)	H21A—C21—H21C	109.5
C7—C8—H8A	109.2	H21B—C21—H21C	109.5
C9—C8—H8A	109.2	C6—C22—H22A	109.5
C7—C8—H8B	109.2	C6—C22—H22B	109.5
C9—C8—H8B	109.2	H22A—C22—H22B	109.5
H8A—C8—H8B	107.9	C6—C22—H22C	109.5
C8—C9—C10	112.73 (17)	H22A—C22—H22C	109.5
C8—C9—H9A	109.0	H22B—C22—H22C	109.5
C10—C9—H9A	109.0	C10—C23—H23A	109.5
C8—C9—H9B	109.0	C10—C23—H23B	109.5
C10—C9—H9B	109.0	H23A—C23—H23B	109.5
H9A—C9—H9B	107.8	C10—C23—H23C	109.5
C23—C10—C9	108.29 (16)	H23A—C23—H23C	109.5
C23—C10—C5	114.92 (15)	H23B—C23—H23C	109.5
C9—C10—C5	107.32 (16)	C13—C24—H24A	109.5
C23—C10—C11	112.61 (16)	C13—C24—H24B	109.5
C9—C10—C11	107.17 (14)	H24A—C24—H24B	109.5
C5—C10—C11	106.16 (14)	C13—C24—H24C	109.5
C12—C11—C2	110.55 (15)	H24A—C24—H24C	109.5
C12—C11—C10	113.19 (15)	H24B—C24—H24C	109.5
C2—C11—C10	116.54 (14)	C15—C25—H25A	109.5
C12—C11—H11	105.1	C15—C25—H25B	109.5
C2—C11—H11	105.1	H25A—C25—H25B	109.5
C10—C11—H11	105.1	C15—C25—H25C	109.5
C11—C12—C13	112.61 (15)	H25A—C25—H25C	109.5
C11—C12—H12A	109.1	H25B—C25—H25C	109.5
C13—C12—H12A	109.1	C17—C26—H26A	120.0
C11—C12—H12B	109.1	C17—C26—H26B	120.0
C13—C12—H12B	109.1	H26A—C26—H26B	120.0
H12A—C12—H12B	107.8	C18—O2—C19	115.72 (16)
C17—C13—C14	111.82 (16)	C15—O5—H1O5	109.5
			
C17—C1—C2—C3	179.65 (14)	C5—C10—C11—C2	−54.13 (19)
C16—C1—C2—C3	59.35 (19)	C2—C11—C12—C13	58.8 (2)
C18—C1—C2—C3	−57.47 (19)	C10—C11—C12—C13	−168.36 (15)
C17—C1—C2—C20	−60.19 (18)	C11—C12—C13—C17	−55.3 (2)
C16—C1—C2—C20	179.50 (15)	C11—C12—C13—C14	63.8 (2)
C18—C1—C2—C20	62.69 (19)	C11—C12—C13—C24	−179.52 (17)
C17—C1—C2—C11	61.04 (17)	C17—C13—C14—O4	−167.3 (2)
C16—C1—C2—C11	−59.27 (18)	C24—C13—C14—O4	−40.0 (3)
C18—C1—C2—C11	−176.09 (15)	C12—C13—C14—O4	76.1 (2)
C20—C2—C3—C4	74.34 (19)	C17—C13—C14—C15	11.4 (2)
C11—C2—C3—C4	−49.46 (19)	C24—C13—C14—C15	138.78 (18)
C1—C2—C3—C4	−166.01 (14)	C12—C13—C14—C15	−105.16 (19)
C2—C3—C4—C5	58.4 (2)	O4—C14—C15—O5	95.3 (2)
C3—C4—C5—C10	−63.8 (2)	C13—C14—C15—O5	−83.44 (19)
C3—C4—C5—C6	161.82 (16)	O4—C14—C15—C25	−26.5 (3)
C4—C5—C6—C7	179.97 (16)	C13—C14—C15—C25	154.76 (18)
C10—C5—C6—C7	49.0 (2)	O4—C14—C15—C16	−153.0 (2)
C4—C5—C6—C21	−62.1 (2)	C13—C14—C15—C16	28.2 (2)
C10—C5—C6—C21	166.93 (17)	O5—C15—C16—O6	−91.8 (2)
C4—C5—C6—C22	59.3 (2)	C25—C15—C16—O6	28.1 (3)
C10—C5—C6—C22	−71.7 (2)	C14—C15—C16—O6	155.85 (18)
C21—C6—C7—O3	15.0 (3)	O5—C15—C16—C1	86.0 (2)
C22—C6—C7—O3	−101.4 (2)	C25—C15—C16—C1	−154.06 (18)
C5—C6—C7—O3	133.6 (2)	C14—C15—C16—C1	−26.3 (2)
C21—C6—C7—C8	−165.2 (2)	C17—C1—C16—O6	163.11 (18)
C22—C6—C7—C8	78.4 (2)	C18—C1—C16—O6	43.8 (2)
C5—C6—C7—C8	−46.6 (2)	C2—C1—C16—O6	−77.6 (2)
O3—C7—C8—C9	−128.7 (2)	C17—C1—C16—C15	−14.7 (2)
C6—C7—C8—C9	51.5 (3)	C18—C1—C16—C15	−134.06 (16)
C7—C8—C9—C10	−55.0 (3)	C2—C1—C16—C15	104.64 (18)
C8—C9—C10—C23	−70.2 (2)	C14—C13—C17—C26	129.3 (2)
C8—C9—C10—C5	54.4 (2)	C24—C13—C17—C26	4.4 (3)
C8—C9—C10—C11	168.11 (17)	C12—C13—C17—C26	−116.5 (2)
C4—C5—C10—C23	−65.9 (2)	C14—C13—C17—C1	−55.9 (2)
C6—C5—C10—C23	66.6 (2)	C24—C13—C17—C1	179.19 (18)
C4—C5—C10—C9	173.62 (15)	C12—C13—C17—C1	58.3 (2)
C6—C5—C10—C9	−53.9 (2)	C16—C1—C17—C26	−128.2 (2)
C4—C5—C10—C11	59.28 (18)	C18—C1—C17—C26	−12.3 (3)
C6—C5—C10—C11	−168.29 (14)	C2—C1—C17—C26	111.7 (2)
C3—C2—C11—C12	−179.43 (15)	C16—C1—C17—C13	57.01 (19)
C20—C2—C11—C12	58.88 (19)	C18—C1—C17—C13	172.94 (15)
C1—C2—C11—C12	−59.47 (17)	C2—C1—C17—C13	−63.13 (18)
C3—C2—C11—C10	49.48 (19)	C17—C1—C18—O1	108.2 (2)
C20—C2—C11—C10	−72.2 (2)	C16—C1—C18—O1	−132.9 (2)
C1—C2—C11—C10	169.43 (14)	C2—C1—C18—O1	−13.4 (3)
C23—C10—C11—C12	−57.4 (2)	C17—C1—C18—O2	−70.5 (2)
C9—C10—C11—C12	61.6 (2)	C16—C1—C18—O2	48.4 (2)
C5—C10—C11—C12	176.01 (14)	C2—C1—C18—O2	167.95 (16)
C23—C10—C11—C2	72.4 (2)	O1—C18—O2—C19	4.0 (3)
C9—C10—C11—C2	−168.58 (16)	C1—C18—O2—C19	−177.29 (18)

### Hydrogen-bond geometry (Å, °)

**Table:**

*D*—H···*A*	*D*—H	H···*A*	*D*···*A*	*D*—H···*A*
O5—H1O5···O3^i^	0.82	2.05	2.870 (2)	173
C23—H23A···O5^ii^	0.96	2.68	3.173 (2)	112

Symmetry codes: (i) *x*+1/2, −*y*+3/2, −*z*+2; (ii) −*x*+3/2, −*y*+2, *z*−1/2.
